# Syphilis testing in blood donors, France, 2007 to 2022

**DOI:** 10.2807/1560-7917.ES.2024.29.32.2400036

**Published:** 2024-08-08

**Authors:** Syria Laperche, Claire Sauvage, Sophie Le Cam, Florence Lot, Lucile Malard, Pierre Gallian, Elodie Pouchol, Pascale Richard, Pascal Morel, Philippe Grange, Pierre Tiberghien, Nadjet Benhaddou, Nicolas Dupin

**Affiliations:** 1 Direction médicale, EFS Siège, Saint Denis, France; 2 Direction des maladies infectieuses, Santé publique France, Saint Maurice, France; 3 Direction nationale, EFS Siège Saint Denis, France; 4 Centre National de Référence des Infections Sexuellement transmissibles, Cochin Hospital, APHP, Paris, France; 5 Université Paris Cité, Institut Cochin-Inserm U1016, Paris, France; 6 Université de Franche-Comté, EFS, INSERM, UMR RIGHT, Besançon, France

**Keywords:** Syphilis, blood donors, diagnostic, screening, epidemiology, France, sexually transmitted infections

## Abstract

**Background:**

Syphilis in blood donors (BD) has increased in many countries.

**Aim:**

We aimed to describe trends in syphilis seroposivity in BD in France, to identify risk factors and assess if a non-treponemic test (NTT) could define BD having recovered from syphilis for more than 1 year.

**Methods:**

The analysis covered the period 2007 to 2022 and 45,875,939 donations. Of the 474 BD syphilis-positive in 2022, 429 underwent additional investigations with an NTT. History of syphilis was obtained at the post-donation interview or based on serology results for repeat donors.

**Results:**

Until 2021, positivity rates remained stable (mean: 1.18/10,000 donations, range: 1.01–1.38). An increased rate was observed in 2022 (1.74/10,000; p = 0.02). Over the whole study period, prevalence was 2.2 times higher in male than in female BD (4.1 times higher in 2022). The proportion of males with an identified risk factor who have sex with men increased from 16.7% in 2007 to 64.9% in 2022. Based on NTT, 79 (18%) of the donors who were seropositive in 2022 were classified as having been infected in the previous year. History of syphilis was available for 30 of them. All had an infection within the previous 3 years. Among seven donors with a syphilis < 12 months before testing, one had an NTT titre ≥ 8, three a titre between 1 and 4, three were negative.

**Conclusion:**

Syphilis seropositivity increased considerably in BDs in 2022, mostly in males, notably MSM. Available data did not allow appropriate evaluation of the NTT to distinguish recent from past infection.

**Key public health message**

**What did you want to address in this study?**

We wanted to assess trends in how many blood donors in France have antibodies indicating a previous syphilis infection. We also wanted to identify risk factors for being syphilis-positive and search for laboratory tests that could distinguish donors who had syphilis within the past year from them whose infection was longer in the past.

**What have we learnt from this study?**

In France, syphilis seropositivity increased substantially in blood donors in 2022, mostly in male donors, notably men who have sex with men; this trend is the same in the general population. We failed to identify an accurate biological diagnostic strategy able to identify a person with a current syphilis infection from one who had recovered from an infection at least 1 year in the past.

**What are the implications of your findings for public health?**

The increasing syphilis rate should encourage the implementation of measures to prevent the transmission in the general population. Further research is needed to improve diagnostic tests so that they can provide information to allow a person with cured syphilis to donate blood.

## Introduction

Syphilis, caused by the spirochete bacterium *Treponema pallidum*, is mostly acquired after sexual contact with an infected individual. Transmission of *T. pallidum* is highest in primary and secondary syphilis, and ca 30% of people will develop syphilis after sexual contact with a person with syphilis lesions. Syphilis can also be transmitted from mother to child during pregnancy. Transmission by transfusion of blood components (excluding plasma) from infected donors have also been described, although such transmission has not been reported for many decades [1,2].

Syphilis evolves through three stages. Primary syphilis occurs within the first 3 months after exposure and usually lasts around 3 weeks with symptoms such as painless lesions and chancres (ulcus durum) in the external anogenital regions, hands or lips. However, chancre is frequently not visible and can heal spontaneously, which often makes difficult to attribute the lesion to syphilis. The spirochetes spread via blood and lymph to different organs, resulting in systemic infection. If the infection is not treated, it can progress to secondary syphilis, which generally occurs 2–8 weeks after the disappearance of the chancre and is characterised by a great variety of symptoms affecting many organs such as the skin (the typical rash/exanthema), central nervous system, liver, kidneys and eyes. After the symptoms resolve, patients can be asymptomatic for many years (latent syphilis). Some patients develop tertiary syphilis, which can affect the brain and nervous system, the heart and blood vessels, the eyes and the ears. Only a minority of untreated individuals with syphilis develop the tertiary stage, as the majority will develop latent syphilis. Syphilis can be asymptomatic and classify as early or late latent syphilis depending on whether the last negative serology before the positive test was less or more than 1 year ago. Successful treatment does not provide immunity, and reinfections can occur, which are frequently asymptomatic and have been reported to be common especially in the population of men who have sex with men (MSM) [3].

Diagnosis of syphilis is currently based on serological methods including two different types of tests: treponemal tests (TT) and non-treponemal tests (NTT). The TT detect antibodies (Ab) directed against antigens that are specific to treponemes. With some exceptions, TT-positive results remain positive throughout an individual’s life regardless of whether the individual is currently infected or has been cured. The NTT detect Ab directed against cardiolipin, a phospholipid found in cell membranes present in a variety of tissues and also in the membrane of *T. pallidum*. Antibodies to cardiolipin, are indirect markers for syphilis but are not specific of this infection. Generally, individuals with syphilis infection have reactive results with NTT, and those who resolve the infection do not remain reactive with NTT for more than 1–2 years after successful treatment. Therefore, NTT are considered as useful in identifying recent syphilis infection and to monitor the progression of syphilis and response to antibiotic therapy. Notably, neither assay (TT or NTT) reliably identifies patients in the very early stage of syphilis before Ab to either treponemal antigens or to cardiolipin have appeared [4].

In Europe, syphilis notifications increased continuously between 2012 and 2021 in the general population, with a marked increase among men, mainly due to an increase in the number of cases among MSM [5]. The rate of syphilis in the blood donor population has also increased in many countries [6-8]. The fact that deferral of MSM from blood donation has gradually been shortened and sometimes replaced by sexual risk assessment irrespective of gender, has been reported as a possible cause of this increase [6,7,9]. In France, MSM deferral for blood donation was reduced in July 2016 from permanent to 12 months since last sexual activity and further reduced to 4 months in April 2020. However, quarantine plasma donation by MSM donors with the same deferral rules as for other donors was introduced in July 2016 and continued up to March 2022 when MSM-specific deferral criteria were removed for all blood donations [10]. 

In this context, and because the majority of syphilis cases with information on transmission were reported in MSM (77% in 2021 according to the European Centre for Disease Prevention and Control [5] and 71% in 2022 in France) [11], we aimed to describe trends in syphilis rates over the past 16 years and to evaluate risk behaviours in syphilis-positive blood donors. In addition, given that the deferral criteria for blood donations make eligible blood donors who have recovered from syphilis for more than 1 year, we investigated whether the addition of an NTT test could distinguish between a recent and a past infection.

## Methods

### Study population

Blood donors included in the study were those who donated between January 2007 and December 2022 at the National Blood Service (Etablissement Français du Sang, EFS) and who were confirmed seropositive for syphilis Ab. A subset of these donors, corresponding to donations collected in 2022, were subjected to additional biological investigations at the National Reference Centre (NRC) for syphilis (see below).

Data for analysis were retrieved from the national epidemiological donor surveillance database, which contains for each confirmed positive donor: date of index donation, date of previous donations, demographical characteristics (age, sex), risk factors and clinical history obtained during the post donation medical consultation. We also retrieved the total number of donations and donors screened classified per status (first time blood donors (FTBD) or repeat blood donors (RBD)).

The syphilis status of the donor was assigned as recent (within 3 years) if the donor indicated a syphilis acquired in the past 3 years at the post-donation consultation, or, according to our haemovigilance procedure, if the last donation given within that 3-year period was confirmed negative when retrospectively tested in the archived sample. The 3-year period was established to match the length of time plasma samples from donations are kept in the biobank.

### Syphilis donation testing

All donations from eligible donors were individually tested for *T. pallidum* Ab with automated haemagglutination tests (Syphagen TPHA AUTO BIOKIT and TPHA Newbio – PK TPHA, BioRad) until the end of 2021. Since the last quarter of 2021, these assays have been progressively replaced by a high-throughput automated chemiluminescence microparticle immunoassay (CLIA) (Elecsys Roche) showing a sensitivity nine times higher than the previous assays (55 international units (IU)/mL vs 6.25 IU/mL, data not shown). All repeatedly reactive samples (i.e. if sample/cut off ratio is ≥ 1) at screening are tested with the ARCHITECT syphilis TP assay (Abbott) and subjected to confirmatory testing using an immunoblot (INNO-LIA syphilis Assay, Innogenetics). Confirmed serology-positive donations are removed from the blood supply and the donors are invited for a post-donation medical interview to identify potential sources of infection. Today, donors with a past history of syphilis, even if successfully treated, are not eligible to donate.

A plasma sample of each confirmed positive donation collected in 2022 was analysed at the NRC for syphilis with a TT (ARCHITECT Syphilis TP assay), an NTT (Launch Diagnostics ASI RPR Card Test, Arlington Scientific, on the automated Gold Standard Diagnostics AIX 1000 RPR analyser) and, when positive with both assays, with an immunoblot (Virotech, Dynablot automatic system). Interpretation criteria adopted by the NRC on biological results are summarised in Table 1.

**Table 1:** Biological interpretation criteria of biological patterns in diagnostic tests established by the National Reference Centre for Syphilis, France, 2007–2022

Treponemal test^a^	Non-treponemal test^b^titre	Immunoblot^c^	Interpretation
Not reactive	Not done	Not done	Negative
Reactive	≥ 8	Positive (or not done)	Active syphilis (< 1 year)
Reactive	1–4	Indeterminate, confirmed (or not done)	Early syphilis or resolved syphilis or endemic treponematosis
Reactive (sample/cut-off > 3)	0	Indeterminate, confirmed (or not done)	Early syphilis or resolved syphilis
Reactive (sample/cut-off ≤ 3)	0	Negative	Non-specific reaction or resolved syphilis
Reactive (sample/cut-off ≤ 3)	0	Indeterminate or positive	Early syphilis or resolved syphilis

^a^ ARCHITECT Syphilis TP assay, Abbott. Reactive when sample/cut-off ratio ≥1.

^b^ Launch Diagnostics ASI RPR Card Test (Arlington Scientific) on the automated Gold Standard Diagnostics AIX 1000 RPR analyser.

^c^ Immunoblot Virotech, (Dynablot Automatic system).

### Incidence rate

Syphilis incidences were calculated among RBD, in five sequential 3-year periods from 2008–2010 to 2020–2022, as the number of donors who converted from negative to confirmed positive in one of the 3-year intervals divided by the total person-years (PY), as previously described [12]. We established seroconversion on the basis of clinical information at the post-donation medical consultation and/or, as required by our haemovigilance procedure, if the archived sample corresponding to the previous negative donation given in the 3-year interval was retrospectively confirmed negative with the CLIA test.

### Statistical analysis

Means were compared using ANOVA test. Trends over time were analysed by using Cochran–Armitage tests. We considered differences between compared groups as statistically significant if p < 0.05. Analyses were performed in SAS 9.4 (SAS Institute).

## Results

### Trends in syphilis antibody results in blood donations from 2007 to 2022

Between 2007 and 2022, among 45,875,939 donations (6,695,847 from FTBD and 39,180,092 from RBD) tested, 5,580 were confirmed syphilis Ab-positive (3,644 and 1,936 in FTBD and RBD, respectively). The mean number of positive BD was 341 (SD +/− 40) between 2007 and 2021 but reached 462 in 2022.

**Figure 1:**
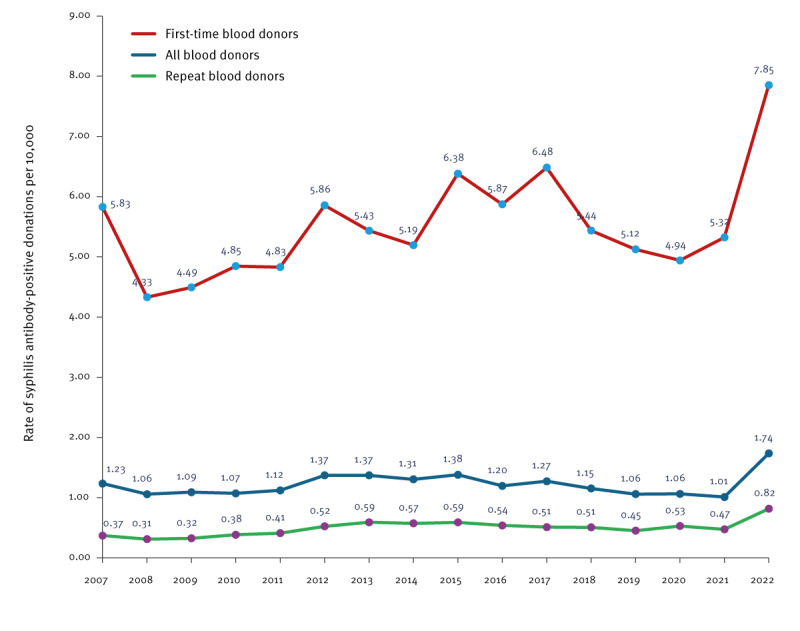
Trends in syphilis antibodies positive rate in blood donors, France, 2007–2022 (n = 5,580)

From 2007 to 2021, the yearly syphilis rate fluctuated, albeit without significant variations (p = 0.3) (Figure 1). In Supplementary Table S1, we provide the number of anti-syphilis antibody-positive donations from 2007 to 2022. The mean positivity rates per 10,000 blood donations were 1.18 (range: 1.01–1.38), 5.36 (range: 4.33–6.48) and 0.47 (range: 0.31–0.59) in all donations, FTBD and RBD, respectively. Increased rates were observed in 2022 (vs the period 2007 to 2021) with 1.74 positive per 10,000 total donations (p = 0.02), and 7.85 and 0.82 per 10,000 in FTBD (p < 0.0001) and RBD (p < 0.0001), respectively (Figure 1).

**Figure 2:**
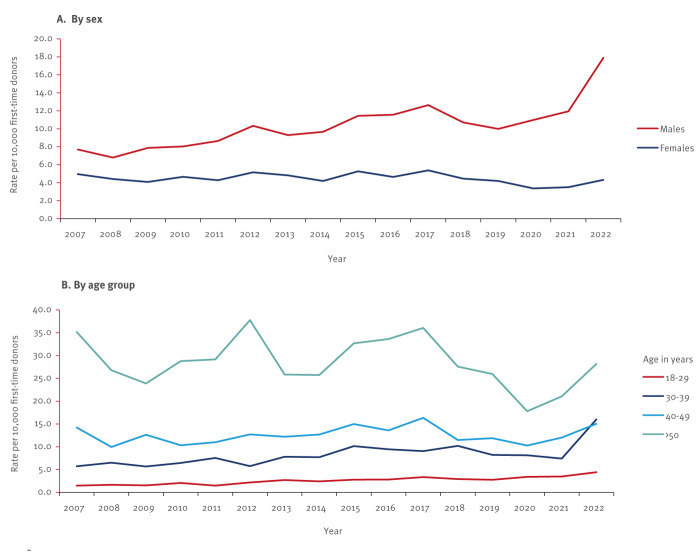
Prevalence of syphilis antibodies per 10,000 donations in first-time blood donors, France, 2007–2022 (n = 3,649)

In first time blood donors, analysis by sex showed that the prevalence was 2.2 times higher in males than in females over the whole study period, while in 2022, prevalence was 4.1 times higher in males than in females (17.9 vs 4.3/10,000 in FTBD). The prevalence remained relatively stable (p = 0.2) in females between 2007 and 2022 (mean: 4.48/10,000, range: 4.09–5.16), while in males, it increased steadily from 7.70/10,000 in 2007 to 17.9/10,000 in 2022 (p < 0,0001) with the exception of a short drop between 2018 and 2020 (Figure 2A). The prevalence increased with age from 2.4/10,000 in 18–29-year-olds to 28.5/10,000 in donors 50 years and older in the whole study period with minor fluctuations. The prevalence increased in all age groups between 2021 and 2022, but particularly in 30–39-year-olds, who had a prevalence two times higher in 2022 than in 2021 (16.0 vs 7.4/10,000) (Figure 2B).

### Risk behaviours

Risk factors were unknown for the vast majority of syphilis-positive blood donors over the study period, even though this proportion decreased from 86.3% (289/335) in 2007 to 53% (251/474) in 2022.

**Figure 3:**
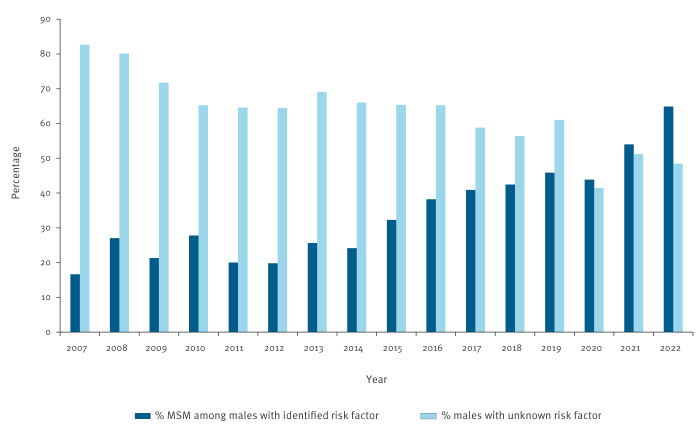
Trends in the percentage of risk factors identified at post-donation interview in syphilis antibody-positive male blood donors, France, 2007–2022 (n = 3,955)

Among men who tested positive for syphilis and for whom a risk factor was declared, the percentage of those reporting having sex with men increased over the period, from 16.7% (6/36) in 2007 to 64.9% (120/185) in 2022 (Figure 3). The majority of women with a known risk factor were infected through heterosexual intercourse.

MSM: men who have sex with men.

### Syphilis incidence in blood donors 

Syphilis incidence per 100,000 PY calculated in five 3-year periods increased from 2.48 (0.56–0.70) in 2008–2010 to 4.66 (0.77–0.92) in 2012–2014 and 5.61 in 2014–2016, and decreased to 4.67 (0.79–0.94) in 2017–2019 and 3.96 (0.74–0.90) in 2020–2022. The majority of cases of recent syphilis (i.e. converted from negative to confirmed positive in the 3-year interval) involved men (81% in 2020–2022 and 83% in 2008–2010) and 35% were aged 18–29 years in the whole study period (39% in 2008–2010).

### Syphilis investigations in seropositive blood donors in 2022

Of the 462 syphilis-positive donations collected in 2022, 429 (93%) were investigated at the NRC. The remaining 33 samples were no longer available. Information on syphilis history was available for 288 of them: 74 (26%) were not aware of having had syphilis, 98 (34%) had syphilis less than 3 years before the donation (seven within the previous year) and 116 (40%) more than 3 years before the donation. Table 2 shows the distribution of samples based on the NRC interpretation criteria and the putative time since infection based on clinical and/or biological information according to the definition described in the *Study population* section. Two donations were found negative at the NCR. The 427 positives were classified into three groups based on NTT titres: samples with an NTT ≥ 8 (n = 79), samples with an NTT between 1 and 4 (n = 108), and NTT-negative samples (n = 240). Of the 79 (18%) that were classified as recent syphilis, information on syphilis history was available for only 30 donors. All of them had an infection within the 3 years before the donation. Conversely, of the 98 donors with a reported syphilis acquired in the 3 years before donation, 68 were deemed not recent based on an NTT titre < 8. Notably, 47 of those had a negative NTT. Among seven donors reporting a syphilis in the 12 months before donation, one had an NTT titre ≥ 8, three a titre between 1 and 4, and three were negative.

**Table 2:** Syphilis-positive donations collected in 2022, by presumed date of infection based on post-donation interview and cardiolipin antibody titre, France, 2007–2022 (n = 429)

Syphilis history	NTT^a^ titre: ≥ 8	NTT^a^ titre: 1–4	NTT^a^: 0	Negative	Total	
					n	%
≤ 1 year	1	3	3	0	7	1.6
>1 year – 3 years	29	18	44	0	91	21.2
> 3 years	0	23	92	1	116	27
Not aware of an infection	27	13	33	1	74	17.2
No information	22	51	68	0	141	32.9
**Total**	**79 (18.4%)**	**108 (25.2%)**	**240 (55.9%)**	**2 (0.5%)**	**429**	

NTT: non treponemal assay.

^a^ Launch Diagnostics ASI RPR Card Test (Arlington Scientific) on the automated Gold Standard Diagnostics AIX 1000 RPR analyser.

These results were consistent with the low reliability of the NTT in distinguishing an infection less than a year old from an older infection when the history of syphilis is unknown.

Among the 429 BD, 181 were RBD. The 74 who had donated in the 3 years before being found positive were retrospectively tested for Ab in the previous negative donation with the Roche CLIA. Of those, 14 (19%) were retrospectively positive, with a mean delay of 423 days between the last negative and the positive donation (median: 408 days, range: 14–1,044). One donor had not been tested; the remaining 13 donors had an NTT that was negative (n = 10) or < 8 (n = 3).

## Discussion

After relative stability between 2007 and 2021, we report a significant increase in syphilis Ab-positive blood donation rates in 2022 in France, especially in first-time donors and in males 50 years and older (data not shown). The increased prevalence in the donor population as well as the higher prevalence in men are consistent with syphilis epidemiology in the general population [11]. However, the increase with age was unexpected and possibly due to resolved syphilis infections in donors who recently became eligible for blood donation (e.g. MSM older than 50 years who had a serological scar from syphilis in the past). All the data for 2023 are not yet available. However, on the basis of the preliminary results, a slight decrease in the number of syphilis-positive blood donors has been observed, although the number is still at a high level.

Conversely, and unsurprisingly, incident cases corresponding to infection of less than 3 years before donation, were more frequent in young donors. Since 2007, the probable mode of contamination may have changed, with an increase in the number of male blood donors reporting sex between men. This could be explained by the gradual easing of selection criteria for blood donations from MSM since 2016 [10]. However, this increase must be interpreted with caution due to the large proportion of donors with unknown risk factors even though this proportion decreased in the last 2 years of the study.

Rising syphilis seropositivity rates in blood donors have also been reported in several other countries [6,7,13]. However, the rate in the blood donor population was 3 times higher in France (1.17 per 10,000 donations) than in the United Kingdom (UK) (0.38 per 10,000) in the period 2016 to 2019 [6], although both countries use similar safety strategies to prevent syphilis-positive donations entering the blood supply, including Ab testing based on a treponemal test as the first line. Progressively shorter MSM time deferrals, implemented in France since 2016, have also been introduced in the UK and elsewhere [6]. Notably, a recent report from Canada showed that changes in MSM deferral policy was not the driving factor in syphilis positivity in blood donations [7].

In 2022, the syphilis Ab-positive prevalence rate in our BD population was the highest ever observed, with 1.74 per 10,000 donations, i.e. 72% higher than in 2021. These findings could be explained by several factors including: (i) an increased number of syphilis cases in the French population, (ii) a highly sensitive testing method (CLIA) used for the blood screening progressively introduced in 2021 which detects low Ab levels that could correspond to a resolved infection, or (iii) a larger number of MSM eligible for blood donation since the MSM-specific selection criteria were abandoned in March 2022. In France, the surveillance of syphilis is based on sentinel networks, which reported an increase in incidence rates of 110% in 2021 and 2022 [11]. In 2022, 91% of syphilis cases were men, and MSM accounted for the vast majority of cases (80% in anonymous testing centres and 71% in general medical practice) [11]. Notably, although early syphilis accounted for 76% in general medical practice in 2022, the frequency in BD was lower. Deferral criteria preventing donors with clinical symptoms to donate and detection of Ab from past and resolved infection are both possible causes of this difference. The retrospective detection of Ab in 19% of previously negative donations from donors who seroconverted within 3 years indicate that a high proportion of positive donations probably were from resolved infections. 

Although transfusion-transmitted syphilis has not been described for many years, the World Health Organization still recommends screening of blood donations for infection [14]. Many factors contribute to the safety of blood, including the control of syphilis in the general population, self-deferral of blood donors who are symptomatic, deferral of donors who report high-risk behaviour for acquiring infection, syphilis Ab testing, the absence of survival of *T. pallidum* in blood during cold storage, and pathogen inactivation applied in some blood components [15-18]. Although a recent experimental study conducted in rabbits showed that treponemes survived in spiked blood components (7 days in whole blood and 6 days in platelets stored at ambient and cold temperatures), the risk of *T. pallidum* transmission by blood products is extremely low [19]. In Australia, it has been estimated that one transmission would occur every 2.8 million transfusions for no syphilis Ab testing and once every 49.5 million transfusions for universal testing [20]. Nowadays, due to the extremely low probability of *T. pallidum* transfusion transmission, syphilis screening in blood donation is mostly used as a surrogate marker for at-risk behaviours. There is debate whether syphilis testing among blood donors should be restricted or abandoned but few countries have reduced or ceased syphilis testing. 

The management of Ab-positive donors remains challenging. According to the European Directive 2002/98/EC, a deferral period of 1 year after the date of a confirmed cured infection must be applied [21]. Given the difficulties of certifying a recovery, which would mean subjecting donors to iterative follow-up, a permanent exclusion is applied in France when a donor is confirmed positive. This precautionary measure may be considered abusive and discriminatory for donors with a past and recovered syphilis history. As treponemal Ab may persist for many years after infection even after treatment, positive TT results do not imply an infectious syphilis. 

In this study, we evaluated a screening strategy including an NTT as an option to identify a recent infection. Unfortunately, we failed to identify an appropriate diagnostic strategy using NTT that can distinguish recent from past infection, as only 30% of donors with a known syphilis acquired within 3 years had an NTT with a titre > 8. Thus, under an algorithm based on TT and NTT, donations that screened positive by CLIA but negative in the NTT may represent early primary syphilis, treated past syphilis infection, latent syphilis of unknown duration, or a false positive reaction.

This study has some limitations. Firstly, even though MSM are a high-risk group, it is difficult to establish with certainty the impact of a change in MSM donor selection criteria on syphilis rates, given that a large number of positive donors did not report risk factors during the post-donation interview. Then, to evaluate a screening strategy able to identify syphilis of less than 1 year, it would have been useful to focus on donors infected within the previous 12 months, however, such information was unfortunately available for only seven donors, and only one had an NTT > 8.

Diagnosis of syphilis remains challenging, especially in absence of clinical manifestations and follow-up. Treponemal assays are more sensitive than NTT in detecting positive specimens, but not all positive donors are infectious as demonstrated by the extremely small proportion of syphilis-positive blood donors who are positive for *T. pallidum* DNA [22-24]. Changes in syphilis laboratory screening approaches are questionable considering that is it not acceptable to impair blood safety and that the presence of *T. pallidum* Ab in blood donors is associated with increased risk of sexually transmitted infections including HIV [8,25].

## Conclusion

We observed a significant increase in syphilis Ab prevalence in blood donors in France in 2022. We were unable to identify an accurate biological diagnostic strategy that would allow distinguishing between recent and past infection. The rehabilitation of blood donors who have recovered from infection more than 1 year before donation could be considered if the laboratory testing options were able to diagnose with a high probability an absence of ongoing infection and including reinfections. Further research is needed to improve diagnostic tests that can more easily identify recovered individuals in order to modify prevention measures for blood safety.

## Supplementary Data

151000 Supplement
