# Supplementary material for: Syphilis testing in blood donors, France, 2007 to 2022
Source: Euro Surveill. 2024 Aug 8;29(32):2400036. doi: 10.2807/1560-7917.ES.2024.29.32.2400036 (PMC11312016; doi:10.2807/1560-7917.ES.2024.29.32.2400036)
Supplement: Supplement [file 24-00036_LAPERCHE_Supplement.pdf]

Supplementary Table S1

This supplementary material is hosted by *Eurosurveillance* as supporting information alongside the article "Syphilis testing in blood donors, France, 2007 to 2022" on behalf of the authors, who remain responsible for the accuracy and appropriateness of the content. The same standards for ethics, copyright, attributions and permissions as for the article apply. Supplements are not edited by *Eurosurveillance* and the journal is not responsible for the maintenance of any links or email addresses provided therein.

Number of total and anti-syphilis antibodies positive donations from first time blood donors (FTBDs), repeat blood donors (RBDs) and all donors, from 2007 to 2022, France.

	First time blood donors			Repeat blood donors			All blood donors		
Year	N donations	N positive	Rate p.10,000	N donations	N positive	Rate p.10,000	N donations	N positive	Rate p.10,000
2007	425 523	248	5.83	2 264 197	84	0.37	2 689 720	332	1.23
2008	519 654	225	4.33	2 277 937	71	0.31	2 797 591	296	1.06
2009	547 611	246	4.49	2 432 436	79	0.32	2 980 047	325	1.09
2010	460 253	223	4.85	2 525 103	97	0.38	2 985 356	320	1.07
2011	503 215	243	4.83	2 625 364	108	0.41	3 128 579	351	1.12
2012	479 825	281	5.86	2 539 860	133	0.52	3 019 685	414	1.37
2013	441 584	240	5.43	2 301 125	136	0.59	2 742 709	376	1.37
2014	442 819	230	5.19	2 353 200	135	0.57	2 796 019	365	1.31
2015	401 173	256	6.38	2 539 194	150	0.59	2 940 367	406	1.38
2016	357 648	210	5.87	2 542 155	137	0.54	2 899 803	347	1.20
2017	374 884	243	6.48	2 562 221	131	0.51	2 937 105	374	1.27
2018	380 818	207	5.44	2 525 723	128	0.51	2 906 541	335	1.15
2019	372 697	191	5.12	2 498 309	113	0.45	2 871 006	304	1.06
2020	337 987	167	4.94	2 453 400	130	0.53	2 791 387	297	1.06
2021	302 446	161	5.32	2 426 079	115	0.47	2 728 525	276	1.01
2022	347 710	273	7.85	2 313 789	189	0.82	2 661 499	462	1.74
